# Factors influencing SARS-CoV-2 infection rate in Belgian nursing home residents during the first wave of COVID-19 pandemic

**DOI:** 10.1017/S0950268822000334

**Published:** 2022-02-22

**Authors:** L. Peckeu-Abboud, E. van Kleef, T. Smekens, K. Latour, S. Dequeker, L. Int Panis, M. Laga

**Affiliations:** 1 Institute of Tropical Medicine (ITM), Antwerp, Belgium; 2 Sciensano, Brussels, Belgium

**Keywords:** Belgian nursing homes, risk factors analysis, SARS-CoV infection rate

## Abstract

In April 2020, Belgium experienced high numbers of fatal COVID-19 cases among nursing home (NH) residents. In response, a mass testing campaign was organised testing all NH residents and staff. We analysed the data of Flemish NHs to identify institutional factors associated with increased SARS-CoV-2 infection rates among NH residents. Cross-sectional study was conducted between 8 April and 15 May 2020. Data collected included demographics, group category (i.e. staff or resident), symptom status and test result. We retrieved additional data: number of beds and staff, type of beds (level of dependency of residents) and ownership (public, private for profit/non-profit institutions). Risk factor analysis was performed using negative binomial regression. In total, 695 NHs were included, 282 (41%) had at least one resident tested positive. Higher infection rate among residents was associated with a higher fraction of RVT beds, generally occupied by more dependent residents (incidence rate ratio (IRR) 1.97; 95% CI 1.00–3.86) and higher staff infection rate (IRR 1.89; 95% CI 1.68–2.12). No relationship was found between other investigated NH characteristics and infection rate among residents. Staff-resident interactions are key in SARS-CoV-2 transmission dynamics. Vaccination, regular staff testing, assessment of infection prevention and control strategies in all NHs are needed to face future SARS-CoV-2 epidemics in these settings.

## Introduction

High rates of morbidity and case fatality related to severe acute respiratory syndrome coronavirus 2 (SARS-CoV-2) outbreaks in nursing homes (NHs) for the elderly have been reported across Europe [1]. In Belgium, the first cases of COVID-19 in the general population were notified from February 2020. By May 2020, Belgium experienced one of the highest numbers of reported fatal COVID-19 cases globally. However, during these first months of the pandemic, all deaths reported among possible COVID-19 cases qualified as COVID-19-related deaths [2]. This broad case-definition partially contributed to the relatively high reported COVID-related death burden in Belgium at that time. Importantly, in these early phases, NHs accounted for more than half (413 deaths/million inhabitants, 51%) of the possible cases reported [1].

In response, Belgium implemented a mass testing campaign among all Belgian NHs in early April. The campaign intended to test all NH residents and staff, hence provide situational awareness and guidance for the national COVID-19 response. These cross-sectional data revealed a 2% and 4% SARS-CoV-2 positivity rate, respectively, among NH staff and residents tested (*N* = 321 and 553) [3].

In addition to the extent, these nation-wide testing efforts highlighted the heterogeneity of SARS-CoV-2 spread among Belgian NHs. Over half of the NHs reported no infections among residents or staff, while others experienced large outbreaks with more than 10 residents infected [4]. To date, institutional characteristics associated with higher infection rates among Belgian NH residents during the early pandemic phase remain unclear. One of the few larger scale studies conducted in Europe, i.e. England, identified reduced transmission rates related to factors such as staff to bed ratio, size of the NH and age of residents [5]. In contrast, large-scale regional studies from the United States and Scotland found no such associations [6, 7]. Furthermore, provision and organisational infrastructure of long-term elderly care vary between countries and regions, making the extrapolation of existing findings to other geographical regions not self-evident.

In this study, we conducted a NH-level analysis on institutional risk factors associated with the proportion of NH residents tested positive for SARS-CoV-2 in the largest Belgian region of Flanders. Hence, we aimed to identify factors contributing to SARS-CoV-2 positivity rate in long-term elderly care settings, in the early phase of the pandemic.

## Methods

### National survey among nursing homes

The COVID-19 testing campaign was implemented between 8 April and 15 May 2020. All three Belgian regions organised an individual campaign for their region. All data were centralised in a national database curated by the Belgian national public health institute Sciensano (https://www.sciensano.be). A detailed description of the national survey's study design has been described elsewhere [8]. In brief, each NH organised staff and resident testing on a single day or over the course of several days (depending on their logistics and capacity available). Nasopharyngeal swabs were collected by occupational physicians of the NHs and real-time-PCR testing was performed. Collected data were available to a medical doctor on a secured platform. All participants were informed about the testing campaign and orally agreed to participate. Here, we focus on data collected from NHs located in the Flanders region, including an additional eight NHs located in the Brussels region that fall under Flanders authority. In Flanders, for logistic reasons (such as test availability), priority in testing was given to facilities (*n* = 55) reporting either the highest percentage of residents with COVID-19-related symptoms, the highest percentage of symptomatic staff, or a combination of both. To still allow for rapid insight in the general COVID-19 situation among Flemish NHs, an additional randomly selected number of NHs (*n* = 30) reporting low or no COVID-19 cases among staff or residents (at the time of selection) were also included in the early April testing phase (8–15 April 2020) [9, 10]. Following this early testing phase, a coordinated and harmonised test strategy for the remaining NHs of the entire region was defined. Data collected included test ID number, age, gender, postal code, group category (i.e. staff or resident), NH name, symptom status at the time of testing and test result [8]. Additional data were made available by Sciensano, and linked to the mass testing data restricted to the Flemish region. These data included NH size (number of beds and number of NH staff members), type of beds available (number of beds for highly care-dependent residents (rust-en verzorgingstehuis, abbreviated to RVT beds), number of beds suitable for non-care-dependent residents (rustoord voor bejaarden, abbreviated to ROB beds) and number of short stay beds) and NH ownership (public institutions, private non-profit institutions and private for-profit institutions).

### Selection of NH included in this analysis

**Fig. 1.**
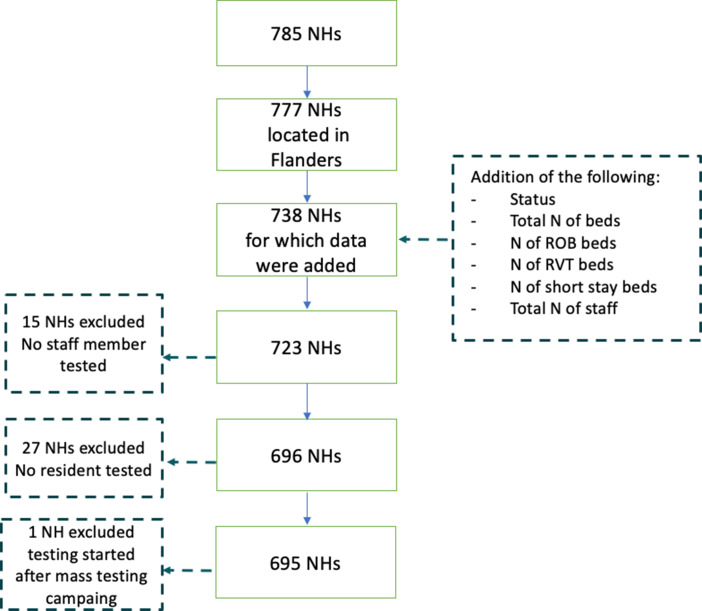
Selection process of nursing homes (NHs) included into the analysis (*n* = 695).

Among all 1542 NHs registered in Belgium, 814 are located in Flanders, 147 in Brussels region (including eight NHs that fall under Flanders’ authority), 573 in Wallonia (including eight that fall under German-speaking Community's authority). Survey methods differed between the three regions, and we analysed data of the largest, i.e. Flemish region, which was made available to us in consultation with the Flemish Public Health Authority. In total, we received data from 785 Flemish NHs, of which 695 were included in our analysis. We excluded NHs for which no staff members or residents were tested, or NHs where testing was conducted outside the scope of the mass testing campaign (Fig. 1).

### Data preparation

We aimed to identify NHs characteristics associated with SARS-COV-2 test positivity rate among residents. To allow for such NH-level analyses, an aggregated database was created. We computed indicators based on the available data and where needed, used guidance from literature (Table 1).

**Table 1.** Characteristics of Flemish nursing homes (NHs) (*n* = 695)

NH characteristics	All NHs (*n* = 695)
Number of beds, *n* (%)	
⩽20 beds	5 (<1)
21–40 beds	35 (5)
41–60 beds	63 (9)
61–80 beds	119 (17)
81–100 beds	135 (19)
>100 beds	338 (49)
Ownership, *n* (%)	
Public institution	179 (26)
Private non-profit institution	385 (55)
Private for profit institution	131 (19)
Type of beds, *n* (%)	
Short stay beds	2207 (3)
ROB beds^a^	31 095 (41)
RVT beds^b^	41 912 (56)
NH with no infection, *n* (%)	324 (47)
NH with 1 or 2 cases, *n* (%)	155 (22)
NH with 3–9 cases, *n* (%)	109 (17)
NH with ≥10 cases, *n* (%)	107 (15)
Ratio NH staff members/residents	0.92 (0.74–1.11)
Residents	
*n* (%)	78 589 (54)
*n* tested (%)	66 209 (51)
*n* tested per NH, median (IQR)	89 (62–117)
Age (in years), median (IQR)	86 (85–87)
*n* tested positive (%)	2492 (4)
*n* asymptomatic and tested positive/*n* tested positive (%)	1896/2492 (76)
NH staff members	
*n* (%)	67 383 (46)
*n* tested (%)	62 989 (49)
*n* tested per NH, median (IQR)	82 (57–116)
Age (in years), median (IQR)	42 (40–44)
*n* tested positive (%)	1430 (2)
*n* asymptomatic and tested positive/ *n* tested positive (%)	1035/1430 (72)

a Beds for non-care-dependent residents.

b Beds for highly care-dependent residents.

Test positivity rate (also called infection rate, is the proportion of staff members and residents that tested positive for SARS-CoV-2) was calculated by dividing the number of residents or NH staff members testing positive by the total number of residents or NH staff members tested during the mass testing campaign per NH, respectively. The proportion of asymptomatic cases (staff or residents) was defined as the number of residents or NH staff members testing positive but presenting no symptoms of an infection divided by the number of residents or NH staff members testing positive (with or without symptoms). The number of total NH beds was divided by 20 and fitted as a continuous variable, similar to [6], allowing for IRRs per 20-bed increase, which, from a clinical perspective, was considered more meaningful. The proportion of RVT beds was defined as the number of RVT beds (beds for highly care-dependent residents) divided by the total number of beds included in the NH. Since the most affected NHs were given priority in the mass testing campaign, we accounted for individual NH testing starting dates in the analyses (i.e. time as a continuous variable). The size of the outbreak was defined according to the Belgian national definition for institutional outbreak size: small outbreak (1 or 2 SARS-CoV-2 cases reported), medium outbreak (3–9 SARS-CoV-2 cases reported) and large (⩾10 SARS-CoV-2 cases reported).

### Statistical analyses

Descriptive analysis on the NHs characteristics was performed, mean and standard deviation (s.d.) were estimated for normally distributed continuous variables; median and interquartile ranges (IQR) were estimated for non-normally distributed variables. Frequencies were described for categorical variables.

We first explored the association between staff and resident positivity rates (infection rates) as a proxy measure for staff and resident interactions by assessing the non-parametric Spearman's correlation. To further identify risk factors for NH-level test positivity rates, we fitted a negative binomial regression model with an off-set term (i.e. the total number of residents per NHs) to the per-NH proportions of residents tested positive. To measure the relative change (in percentage) between positivity rate among residents and staff members, the staff members' positivity rate was log-transformed. Univariate and multivariate incidence rate ratios (IRRs) and their 95% confidence intervals (CIs) were estimated. Univariate analyses were performed on computed NH characteristics. NH characteristics with a *P*-value ⩽ 0.25 were included in the multivariate analysis. In the multivariate analysis, a *P*-value ⩽ 0.05 was considered significant.

All analyses were performed using R version 3.6.3, using *tidyverse* and *ggplot* packages for our exploratory analyses, the *MASS* package was used for regression model fitting. Scripts are accessible via the GitHub repository: https://github.com/Laureneitm/Belgian_NH_analysis

The study was approved by the Institutional Review Board of the Institute of Tropical Medicine.

## Results

### Characteristics of the NHs and their population

A total of 66 209 (84%) residents and 62 989 (93%) NH staff members were tested during the testing campaign among 695 NHs included in our study. Nearly half of these NHs (*n* = 338, 49%) were large NHs (>100 beds), with the majority of beds (56%) comprising of ‘RVT beds’. NHs were largely private non-profit institutions (*n* = 385, 55%) (Table 1).

During the mass testing campaign, 324 (47%) of NHs did not report a single SARS-CoV-2 infection among residents. Among the NHs that reported positive cases, the majority (*n* = 155, 22%) reported one or two SARS-CoV-2 cases (a small outbreak according to the Belgian national definition for institutional outbreak size). This was followed by 17% (*n* = 109) and 15% (*n* = 107) of NHs reporting medium (3–9 cases) and large (⩾10 cases) outbreaks, respectively. Per NH, a median of 89 residents (IQR 62–117) and 82 (IQR 57–116) NH staff members were tested on average. Among NH staff members, the median age was 42 years (IQR 40–44), compared to 86 years (IQR 85–87) among residents. Among the residents and NH staff members that tested positive, high percentages of asymptomatic cases were reported (76% and 72%, respectively, Table 1).

### SARS-CoV-2 infections among NH staff and residents by institution

**Fig. 2.**
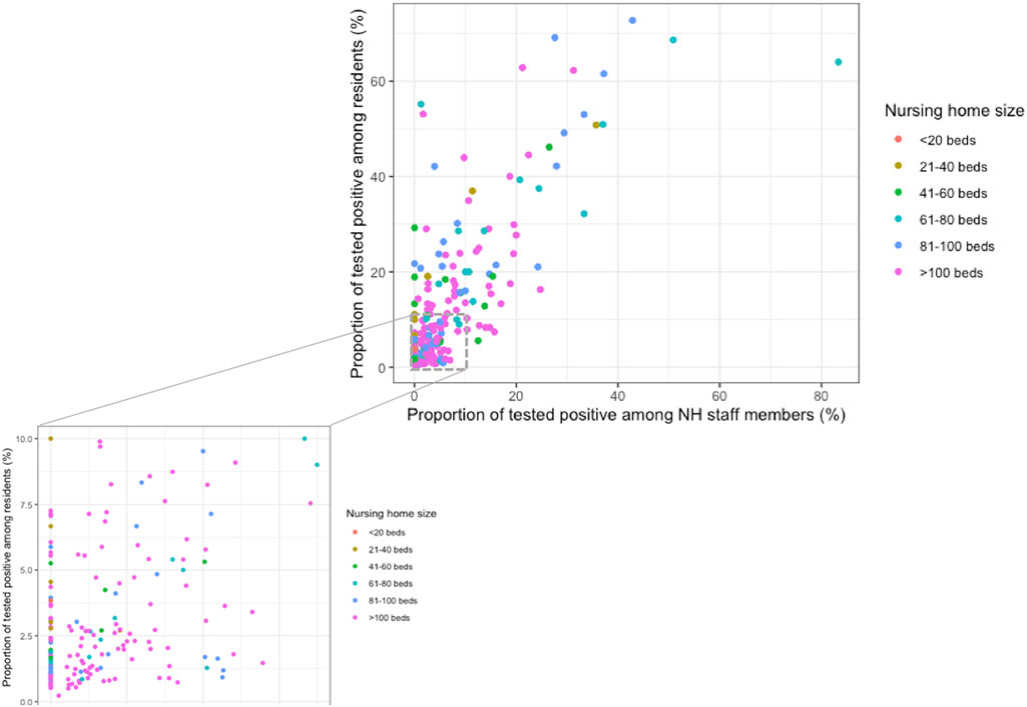
Proportion of tested positive among residents and NH staff members in the 282 Flemish nursing homes (NHs) with a proportion of tested positive residents above 0 (Spearman's correlation coefficient = 0.63, *P*-value<0.001).

For the 282 (41%) institutions with ⩾1 infected residents reported, the infection rates varied from 0.2% to 72% (median of 4%) among residents and from 0% to 83% among NH staff members (median of 2%) (Fig. 2). We found a significant positive correlation between the proportion of staff members and residents tested positive, respectively (Spearman's correlation coefficient = 0.63, *P*-value < 0.001). The majority of NHs with ⩾1 cases concerned large NHs (*n* = 174, 62%). Nonetheless, on visual inspection, reported infection rates among staff and/or residents did not appear to relate to NHs size, which was further explored in the multi-variate analysis (Fig. 2).

### Risk factor analyses on the proportion of tested positive residents

We considered nine putative NH characteristics for increased SARS-CoV-2 positivity among NH residents at the NH level (Table 2). Following our univariate analyses, we included the proportion of RVT beds (a proxy for the degree of resident dependency), resident and staff age, and the proportions of staff tested positive and asymptomatic cases among those tested positive (for both residents and NH staff) in a multivariate regression model.

**Table 2.** Incidence rate ratio (IRR) (univariate and multivariate negative binomial regression) of associations between the proportion of tested positive among residents and nursing home (NH) characteristics

NH characteristics	Univariate analysis		Multivariate analysis^a^	
	IRR (95% Cl)	*P*-value	IRR (95% Cl)	*P*-value
*n* total beds (=total population of resident) (per 20 beds) (0 missing value)	1.01 (0.95–1.08)	0.86	–	–
Proportion of RVT beds^b^ (*n* RVT beds/*n* total beds) (0 missing value)	2.95 (1.16–7.03)	0.02	1.97 (1.00–3.86)	0.05
Ratio NH staff members/residents (0 missing value)	1.28 (0.75–2.29)	0.37	–	–
Mean age NH staff members (in years) (0 missing value)	1.06 (1.02–1.12)	0.02	1.02 (1.00–1.05)	0.26
Mean age residents (in years) (0 missing value)	0.97 (0.90–1.02)	0.12	1.03 (1.00–1.08)	0.07
Test positivity rate among NH staff members (0 missing value)	1.97 (1.85–2.10)	<0.001	1.89 (1.68–2.12)	<0.001
Proportion of asymptomatic NH staff members (*n* asymptomatic + tested positive NH staff members/*n* positive NH staff members) (0 missing values)	0.40 (0.19–0.82)	0.001	0.85 (0.58–1.25)	0.41
Proportion of asymptomatic residents (*n* asymptomatic + tested positive residents/*n* positive residents) (0 missing values)	0.19 (0.10–0.35)	<0.001	1.05 (0.63–1.72)	0.82
Ownership (0 missing value)	1.06 (0.80–1.41)	0.68	–	–

a Degrees of freedom = 195.

b Beds for highly care-dependent residents.

The multivariate analysis (model fitted on 196 NHs) revealed a positive association between the proportion of residents and staff tested positive (IRR 1.89; 95% CI 1.68–2.12 for each per cent increase). Importantly, a one unit increase in the proportion of RVT beds was associated with a twofold increase in the proportion of residents tested positive (IRR 1.97; IQR 1.00–3.86). No significant association was found for the other NH characteristics.

## Discussion

In this study, we explored NH characteristics that may have predisposed NHs in Flanders to SARS-CoV-2 outbreaks during Belgium's first wave of the COVID-19 epidemic. The mass testing campaign revealed that over half (53%) of Flemish NHs had at least one SARS-CoV-2 infection identified among NH residents and staff, with 15% of NHs facing large outbreaks (⩾10 cases). The risk of infection among NH residents was significantly increased with an increased infection rate among NH staff members and a higher fraction of RVT beds. No associations were found between SARS-CoV-2 positivity rates and size of the NH expressed in number of beds, type of NH (public or private), mean age of the residents and staff, or proportion of asymptomatic tested positive cases among residents and staff.

Both the associations of higher staff infection rate as well as a higher ratio of RVT beds likely point to a key role of interactions between staff members and residents in SARS-CoV-2 transmission dynamics in NH settings. For the latter, RVT beds or rooms are generally occupied by residents requiring higher levels of care, and therefore requiring more staff contact, whilst contact with other residents is limited. Available stock of personal protective equipment (PPE) as well as training on their proper use where limited in the early phase of the pandemic in Belgium. Frontline reports revealed that the lack of equipment and clear infection prevention and control (IPC) strategies (especially testing and staff cohorting) resulted in increased risk of onward SARS-CoV-2 transmission among staff and residents [11]. Along with these results, Telford *et al.*, investigated among a sample of 23 NHs in Georgia 33 key indicators from five IPC categories (hand hygiene, disinfection, social distancing, PPE, and screening for symptoms and elevated temperature) and found that differences between higher- and lower-prevalence NHs occurred most frequently in the social distancing and PPE categories [12]. In our study, we did not have access to reliable data on the availability and use of PPE, nor IPC strategies implemented. Nonetheless, lack of PPE supply and suboptimal screening and testing strategies were universal across Belgian NHs [13].

We found that 47% of the NHs in Flanders did not have any SARS-CoV-2 infection detected in the early April–May 2020 period, implying a marked fraction of NH staff members and residents remained vulnerable to SARS-CoV-2 infections. Similar observations were made in regional studies from North America and Europe, where a large proportion of NHs were still susceptible to SARS-COV-2 infections after May 2020 [6, 14–16]. Post Belgium's testing campaign, several initiatives were taken in Flanders to overcome the COVID-19 crisis in NHs. Strategies to exchange personnel between different settings including hospitals and NHs were implemented [17] and support was provided on medical expertise, training, material and medication. As well as organisation of material supply and deliveries such as PPE within Flemish NHs [18]. Despite these investments, during the autumn second wave 2020, 73% and 49% of Belgian NHs reported ⩾2 and ⩾10 confirmed cases, respectively [19] and at the moment of writing, 96% of Belgian NHs reported ⩾2 possible or confirmed cases [20]. SARS-CoV-2 transmission dynamics and IPC in general in NHs come with a multifactorial set of challenges different from more controllable clinical settings, such as adherence and understanding of IPC guidelines from the residents themselves [21].

In addition, mitigating staff-resident transmission risk in the early phase of the pandemic, as well as over the course of the pandemic, has been challenged by the recognised role of asymptomatic and pre-symptomatic carriers in the SARS-CoV-2 transmission [22–24]. In an earlier analysis of the (national) mass testing campaign data, Hoxha *et al.* concluded that high levels of identified a- and pre-symptomatic carriers (means of 74% and 75% of infected staff and residents, respectively) point towards non-symptomatic individuals having comprised an important driver of transmission within Belgian NHs. Our analyses did not reveal an increased NH-level resident infection rate related to the proportion of asymptomatic infections among staff or residents. However, this may be a result of the limited variation in asymptomatic carriage across all participating NHs (median = 100% (IQR 59–100) and 100% (71–100) for staff and residents, respectively). Moreover, due to the cross-sectional nature of our study, onward transmission could not be established, nor were CT-values available to determine what proportion of asymptomatic carriers was likely ‘infectious’. Longitudinal studies and seroprevalence surveys on staff and residents could enhance our understanding of SARS-CoV-2 transmission in NHs.

While the above underlines the critical importance of good IPC practices; the availability of effective vaccines against COVID-19 has made active immunisation of staff and residents an essential part of infection prevention strategies in long-term care. In Belgium, vaccination campaigns started in December 2020. All residents and staff of the Belgian NHs were offered vaccination. By 24 March (end of the vaccination campaign in NHs), the vaccine coverage among staff varied by region, with 86% in Flanders, 58% in Wallonia and 47% in Brussels [25]. The relative low acceptance among healthcare staff working with the most vulnerable population has initiated debates on compulsory vaccination for healthcare professionals. Our work reinforces the idea that maximum protection (by reaching and sustaining high immunisation coverage rates) and prevention through vaccination among staff of NHs is essential. Compulsory vaccination may be effective in preventing disease outbreaks, but it may also lead to negative vaccination attitudes decreasing vaccination uptake [26, 27]. Governments should take such factors in consideration when implementing vaccination policies.

We found no association between the number of beds (with potentially higher number of residents exposed), and the infection rate among residents. This is in line with two studies from Scotland and Canada involving, respectively, 189 care homes and 618 NHs, where no relationship between care home number of beds and SARS-CoV-2 incidence was detected [6, 28]. Moreover, Abrams *et al*. found that larger facility size was significantly related to the probability of having a COVID-19 case (OR 6.52, *P* < 0.001, for large facilities *vs.* small ones) [7]. In contrast, Shallcross *et al*. found higher occupancy to affect SARS-CoV-2 infection rates in NHs settings in England [29]. Therefore, evidence on the role of NH size in transmission dynamics of SARS-CoV-2 remains unclear and potential confounders (e.g. number of beds per room) could be more informative on the relationship between NH size and SARS-CoV-2 epidemics.

It is well known that older ages result in immune senescence, making elderly care residents vulnerable to emerging infectious diseases such as SARS-CoV-2 [30]. Shallcross *et al.* reported a significant association between increasing age and the odds of infection in residents (aOR 1.01; 95% CI 1.01–1.03) [5]. Belgium's elderly care resident population is relatively old, and average length of NHs stay is about 2 years [31]. The limited age range in Belgian NHs may be a potential explanation for age not constituting a risk factor to SARS-CoV-2 infection rates in our study.

Our study has several strengths. We are among the few studies that used large-scale survey data including 695 NHs, representing 85% of all the NHs located in Flanders (*n* = 814). Additionally, we linked the mass testing data to routinely collected data hence providing relevant insights on the role of NH characteristics. While the survey was implemented with the aim to guide the national response strategy and its design, some limitations have to be recognised. For facilities with more staff and residents than the number of tests assigned, the prioritisation was left at the discretion of the structure. Therefore, it is possible that previously identified infections among residents (before the mass testing campaign) were not tested at the time of the mass testing campaign and were therefore not included in our analysis. This could have led to an underestimated proportion of residents tested positive in some NHs. Furthermore, known cases of staff members in quarantine, not present at the NH during the days of the mass testing campaign were not tested. Therefore, infection rate among staff could be underestimated as well. The number of variables that could be explored was limited to the variables collected during this mass testing campaign. Variables such as isolation procedures for residents, staff cohorting and the number of staff working at different sites could have been potential factors influencing the proportion of positive tests. In addition to the quantitative study, qualitative data could have improved our understanding of the situation that each NH faced, giving more detailed information on limitations in the response capacity.

In conclusion, our analysis showed that two factors (positivity rate among staff and fraction of RVT beds) both related to staff-resident interactions influenced the infection rate of SARS-CoV-2 among residents in the Flemish NH during the first wave of the pandemic. Reducing the impact of COVID-19 in NH settings goes along with an adequate protection of staff members preventing transmission from and to the residents. We recommend the implementation of targeted interventions such as vaccination, regular staff testing, assessment of IPC strategies in all NHs to be able to face future (SARS-CoV-2) epidemics in these settings.

## Data Availability

Data are available on request from the corresponding author, subject to ITM and AZ&G disclosure controls to prevent identification of individuals.
